# COVID-19 with cystic features on computed tomography: A case report

**DOI:** 10.1097/MD.0000000000020175

**Published:** 2020-05-01

**Authors:** Kefu Liu, Yuanying Zeng, Ping Xie, Xun Ye, Guidong Xu, Jian Liu, Hao Wang, Jinxian Qian

**Affiliations:** a Department of Medical imaging; b Intensive Care Unit; c Department of Cardiology; d Department of Respiratory and Critical Care Medicine, The Affiliated Suzhou Hospital of Nanjing Medical University, Suzhou, Jiangsu; e Department of Radiology, Minhang Hospital, Fudan University, Shanghai, China.

**Keywords:** case report, COVID-19, CT, cyst

## Abstract

**Rationale:**

The cystic features of the novel coronavirus disease 2019 (COVID-19) found on computed tomography (CT) have not yet been reported in the published literature. We report the cystic chest CT findings of 2 patients confirmed to have COVID-19-related pneumonia.

**Patient concerns:**

A 38-year-old man and a 35-year-old man diagnosed with severe COVID-19 pneumonia were admitted to the intensive care unit.

**Diagnoses:**

Chest CT findings showed multiple cysts in ground-glass opacities (bilaterally) with/without pneumothorax. The cysts had a smooth inner wall.

**Interventions:**

The patients continued to be given oxygen by mask and received antitussive, phlegm-dispelling treatment.

**Outcomes:**

At follow up, there was a reduction in the number of multiple cystic lesions on CT. To date, 1 patient was discharged from hospital, while the other had been transferred to the rehabilitation department.

**Lessons:**

COVID-19 may independently result in pulmonary cyst formation and pneumothorax; the application of a ventilator may be another causative factor.

## 1 Introduction

The typical chest computed tomography (CT) findings of the novel coronavirus disease 2019 (COVID-19) appear as multiple patchy, ground-glass opacities that progress to or co-exist with bilateral consolidations in multiple lobes and with peripheral distribution^[1–3]^; however, the cystic features of COVID-19 on CT have not yet been reported in the literature. Here, we report the chest CT findings of cystic lesions in 2 patients confirmed to have COVID-19 pneumonia. Of note, written informed consent was obtained from both patients to publish this case report.

## 2 Case report

### 2.1 Case 1

**Figure 1:**
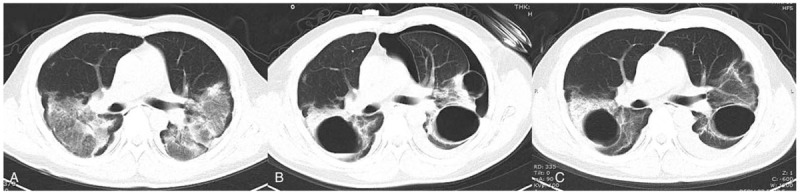
A 38-yr-old man diagnosed with severe COVID-19 pneumonia. (A) The primary CT scan showed bilateral, patchy, ground-glass opacities with co-existing consolidations. (B) Nine days later, a follow-up CT scan showed multiple cysts in the ground-glass opacities (bilaterally) and a ∼20% left pneumothorax. The cysts featured a smooth inner wall; the maximum diameter of these cysts was about 5 cm. (C) The third CT scan (performed 5 d after the second CT scan) showed that the left pneumothorax and a small cyst on the left pulmonary margin had disappeared; the remaining cysts reduced in size slightly.

A 38-year-old man diagnosed with severe COVID-19-related pneumonia (6 days) was admitted to the intensive care unit. The primary CT findings showed bilateral, patchy, ground-glass opacities with co-existing consolidations (Fig. 1A). The patient presented with shortness of breath, chest pain, 92% percutaneous oxygen saturation, and 26 breaths/minute; the patient was thus given 5 L/minute of oxygen by mask. However, 26 days later, follow-up CT findings showed multiple cysts in the ground-glass opacities (bilaterally) and the development of a ∼20% left pneumothorax. The cysts had a smooth inner wall and the maximum diameter of these cysts was ∼5 cm (Fig. 1B). At this time, real-time fluorescent polymerase chain reaction of the patient's sputum was negative for COVID-19 nucleic acid. The patient continued to be given 4 L/minute of oxygen by mask, as well as an antitussive, phlegm-dispelling treatment. The third CT scan, which was performed 5 days after the second CT scan, showed that the left pneumothorax and 1 small cyst on the left pulmonary margin had disappeared; the remaining cysts were slightly reduced in size (Fig. 1C). The patient was discharged from the hospital 21 days after the third CT scan.

### 2.2 Case 2

**Figure 2:**
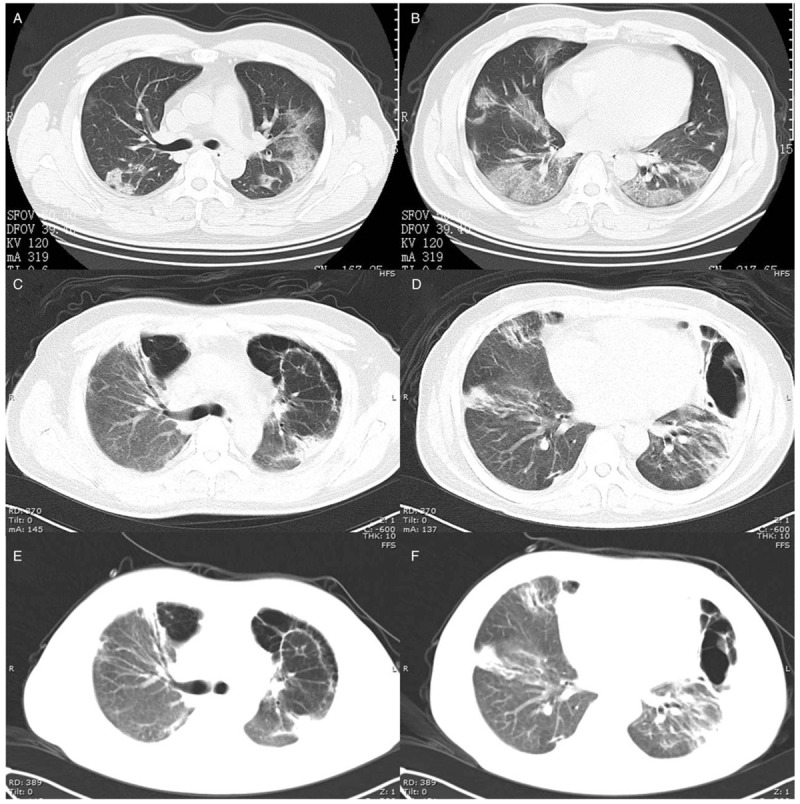
A 35-yr-old man diagnosed with severe COVID-19 pneumonia. (A and B) The primary CT scan showed bilateral, patchy, ground-glass opacities and consolidations. (C and D) Forty days later, a follow-up CT scan showed bilateral, patchy consolidations, and multiple cystic lesions (bilaterally) with peripheral distribution. (E and F) The multiple cystic lesions were found to be slightly reduced in size on the third CT scan, which was performed 2 d after the second CT scan.

A 35-year-old man diagnosed with severe COVID-19 pneumonia (9 days) was admitted to the intensive care unit. The primary CT findings showed bilateral, patchy, ground-glass opacities and consolidations (Fig. 2A and B). The patient developed acute respiratory distress syndrome (ARDS) and type I respiratory failure; as such, the patient was given assisted respiration via a noninvasive ventilator (the inspiratory positive airway pressure ranged from 20 to 26 cm H_2_O; the expiratory positive airway pressure ranged from 5 to 10 cm H_2_O), as well as anti-infective, antivirus treatment. Forty days later, the CT scan findings showed bilateral, patchy consolidations, and multiple cystic lesions (bilaterally) with peripheral distribution (Fig. 2C and D). At present, real-time fluorescent polymerase chain reaction of the patient's sputum was negative for COVID-19 nucleic acid. To treat the patient's paroxysmal cough, which featured a little white mucus, as well as to address the patient's 97% percutaneous oxygen saturation with 19 breaths/minute, the patient's antitussive, phlegm-dispelling treatment was continued. Two days later, the multiple cystic lesions were slightly reduced in size on follow-up CT (Fig. 2E and F). At the time this paper was submitted for publication, the patient had been transferred to the rehabilitation department.

## 3 Discussion

To date, the papers published on COVID-19 have not discussed the presence of cystic lesions in association with this disease. In both of our cases, the pulmonary cystic lesions of COVID-19 were found on CT.

The pulmonary cystic lesions and pneumothorax that were found may have been complications associated with mechanical ventilation.^[4,5]^ The incidence of barotrauma in patients with ARDS following ventilation therapy was 6.5%.^[6]^ Most ventilation-associated cysts were small (<1 cm in diameter) and featured a thick wall with no appreciable internal structure, and were found in a subpleural location.^[7]^ However, some of the literature reported that ARDS might independently result in cyst formation.^[7,8]^ The pulmonary cysts were observed in severe acute respiratory syndrome patients who received only short-term, low-pressure, and volume ventilation or received no mechanical ventilation at all.^[7]^ The reasons for this might include the development of ischemic parenchymal damage, lung fibrosis, low lung compliance, and inflammatory exudate in the airway.^[7–9]^

The pulmonary cysts and pneumothorax that were observed in both of our cases occurred more than 30 days after symptom onset; both patients were in the intermediate and late stages of ARDS, which is when fibrous processes begin to appear.^[10]^ Case 1 did not receive mechanical ventilation therapy; therefore, we speculate that ischemic parenchymal damage, lung fibrosis, and low lung compliance may have led to the formation of cysts and the development of a pneumothorax. Case 2 received a noninvasive ventilator; thus, the application of the ventilator may have constituted an additional reason for the formation of cysts and the development of a pneumothorax.

Recent histological examinations reported bilateral diffuse alveolar damage with cellular fibromyxoid exudates in COVID-19.^[11]^ Pulmonary cystic lesions may form in response to cellular fibromyxoid exudates, which form a valve in the bronchus. Since these lesions cause emphysema and bullae, phlegm-dispelling treatment options may have been more appropriate and effective for both of our cases.

In conclusion, COVID-19 may independently result in pulmonary cyst formation and the development of a pneumothorax; however, the use of a ventilator to manage the symptoms of this disease may be another reason underlying the formation of these lesions in patients affected by this virus.

## Acknowledgments

We thank our colleague, Xinyi Wang, for helpful discussions in article revision. English-language editing of this manuscript was provided by Journal Prep Services.

## Author contributions

**Data curation:** Kefu Liu, Yuanying Zeng, Ping Xie, Xun Ye, Guidong Xu, Jian Liu, Jinxian Qian.

**Funding acquisition:** Kefu Liu.

**Writing – original draft:** Ping Xie, Yuanying Zeng, Hao Wang.

**Writing – review & editing:** Kefu Liu, Jinxian Qian.
